# Protocol for isolating viable human central nervous system T cells

**DOI:** 10.1016/j.xpro.2026.104464

**Published:** 2026-03-25

**Authors:** Hendrik J. Engelenburg, Joost Smolders, Inge Huitinga, Jörg Hamann, Cheng-Chih Hsiao

**Affiliations:** 1 Neuroimmunology Research Group, Netherlands Institute for Neuroscience, Amsterdam 1105 BA, the Netherlands; 2 Department of Immunology, MS Center ErasMS, Erasmus MC, University Medical Center Rotterdam, Rotterdam 3015 GD, the Netherlands; 3 Department of Neurology, MS Center ErasMS, Erasmus MC, University Medical Center Rotterdam, Rotterdam 3015 GD, the Netherlands; 4 Swammerdam Institute for Life Sciences, Center for Neuroscience, University of Amsterdam, Amsterdam 1098 XH, the Netherlands; 5 Netherlands Brain Bank, Netherlands Institute for Neuroscience, Amsterdam 1105 BA, the Netherlands; 6 Department of Experimental Immunology, Amsterdam Institute for Immunology and Infectious Diseases, Amsterdam University Medical Center, Amsterdam 1105 AZ, the Netherlands

**Keywords:** Cell isolation, Flow Cytometry, Immunology, Neuroscience

## Abstract

Brain-resident T cells act as sentinels, monitoring and supporting immune surveillance and homeostasis. Here, we present a rapid protocol for isolating viable T cells from *post-mortem* human brain tissue. We describe the process of extracting cells from multiple CNS compartments—including choroid plexus, leptomeninges, dura mater, cerebrospinal fluid, and parenchyma—as well as matched peripheral blood. We detail steps for achieving this through mechanical and enzymatic tissue dissociation, followed by density gradient centrifugation to isolate mononuclear cells for downstream applications.

For complete details on the use and execution of this protocol, please refer to Hsiao et al*.*^1^

## Highlights

• Rapid isolation of viable mononuclear cells from *post-mortem* human brain tissue
• Guidance on tissue dissociation using enzymatic digestion for different applications
• Instructions for paired assessment of different CNS compartments and blood

## Abstract (editor highlights)

Publisher’s note: Undertaking any experimental protocol requires adherence to local institutional guidelines for laboratory safety and ethics.

## Graphical abstract

**Figure:**
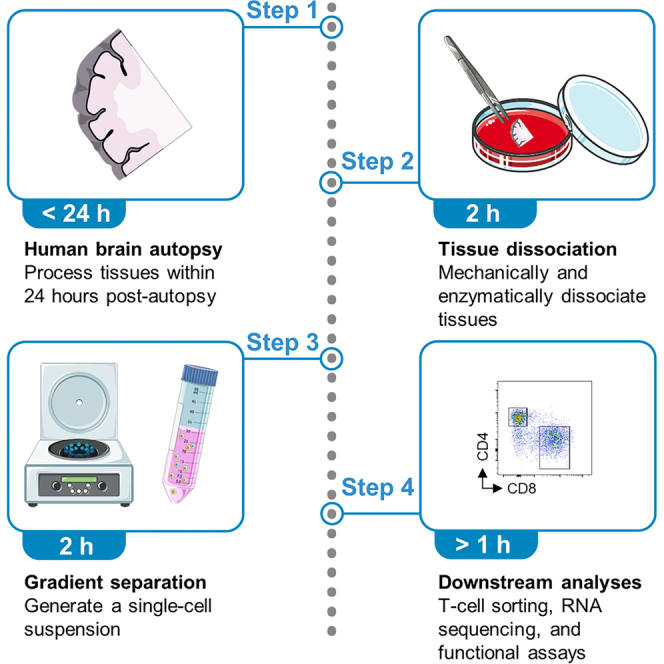

## Before you begin

The protocol described here facilitates the isolation of immune cells from rapid *post-mortem,* autopsy-acquired human central nervous system (CNS) tissues. Isolation is particularly challenging due to the high lipid content of the brain, the low frequency of immune populations, and the rapid degradation *post-mortem*. It outlines the steps for isolating T cells from multiple CNS compartments—including parenchyma (white and gray matter), cerebrospinal fluid (CSF), leptomeninges, dura mater, and choroid plexus—as well as matched peripheral blood as established in our previous work.^1^^,^^2^^,^^3^^,^^4^^,^^5^ This protocol is optimized for T cell isolation and next-generation sequencing. Alternatively, this protocol can also be applied to isolate B cells or myeloid cells, such as macrophages and microglia, as described in earlier studies.^6^^,^^7^^,^^8^

### Timing and tissue quality

The procedure must be performed as soon as possible after autopsy, preferably within 24 h while the tissue is kept in Hibernate A medium at 4°C in the refrigerator immediately after autopsy. To minimize degradation, all tissues, buffers, and cells must be kept cool at 4°C on ice throughout the process. A delay in tissue processing beyond the 24-h window may result in lower yields, reduced RNA integrity, and an increased risk of technical artifacts that may confound downstream results. Since the CSF pH reflects tissue quality, RNA integrity, and the brain donor’s agonal state,^6^ we recommend using tissue where the CSF pH is greater than 6.0.

### Use of inhibitors

Since enzymatic digestion at 37°C can induce temperature-induced transcriptional artifacts,^9^ we have incorporated the use of transcription and translation inhibitors in this protocol. Note that the use of these inhibitors restricts the application of isolated cells in functional assays as they lock the cell state. Additionally, the protocol omits serum during isolation to prevent serum-induced activation of immune cells. The process includes flow-cytometric cell sorting to enable cell profiling and functional studies.

### Biosafety

Since the tissue may carry pathogenic vectors such as blood-borne pathogens or prions, it is critical to work within a Biosafety Level 2 (BSL-2) laboratory and follow Personal Protective Equipment (PPE) guidelines. Tools for tissue dissociation must be sterilized after use, and human tissue waste should always be disinfected with bleach in accordance with institutional waste management policies. Although the tissue is not sterile after autopsy, proper laboratory techniques should be employed in order to minimize potential other sources of contamination, especially if the cells are intended for subsequent culture.

### Innovation

This protocol advances human brain immune cell isolation by establishing a workflow for the parenchyma and biologically critical CNS-border tissues—including choroid plexus, leptomeninges, and dura mater—which represent key interfaces for neuro-immune interaction. By utilizing divergent density gradients (Percoll™ for myelin-rich parenchyma and 25% BSA for low-myelin border samples), this method optimizes cell recovery and viability across distinct anatomical niches, overcoming previous limitations of high lipid interference and low yield in heterogeneous samples.

To address traditional limitations in transcriptomic fidelity, we incorporated a transcription and translation inhibitor cocktail that prevents temperature-induced artifacts and *ex vivo* activation during enzymatic dissociation, ensuring high-fidelity data for next-generation sequencing. This optimization addresses a major drawback of previous methods where the 37°C incubation triggered artificial gene expression, thereby preserving the *in vivo* state of the immune cells.

By standardizing success through a strict 24-h *post-mortem* interval and a CSF pH threshold of > 6.0, this optimized method significantly enhances the reproducibility and accuracy of primary CNS immune cell profiling compared to previous approaches. These specific quality control benchmarks ensure more reliable results for further applications, consistently achieving high cell viability and robust recovery across different anatomical compartments.

### Institutional permissions

Human brain tissues and paired peripheral blood were provided by the Netherlands Brain Bank (Amsterdam, The Netherlands; https://www.brainbank.nl). Informed consent for performing autopsy and utilizing tissue and clinical data for research purposes was obtained from all donors. All procedures were approved by the Ethics Committee of Amsterdam UMC (Location VUmc, Amsterdam, The Netherlands), an Institutional Review Board registered with the U.S. Office of Human Research Protections (IRB number: IRB00002991; Federal-wide Assurance number: 00003703).

***Note:*** It is critical to ensure that appropriate institutional permissions for working with human brain samples are obtained before proceeding with this protocol.

### Preparation of reagents

**Timing: 1 h**

This section outlines the preparation of essential buffers and stocks. For final concentrations and specific volumes, refer to the “materials and equipment” section.

1. Preparation of 25% bovine serum albumin (BSA):
  a. Add 25 g of BSA to a flask and top up to 100 mL with Hanks’ Balanced Salt Solution (HBSS), no calcium, no magnesium, no phenol red.
  b. Dissolve at 4°C.
    ***Note:*** BSA dissolves slowly; prepare this solution well in advance. Do not stir or shake, as BSA foams easily, which can interfere with its performance in density gradient centrifugation. Instead, occasionally invert the container gently until the powder is fully dissolved.
  c. Filter through a 0.2 μm filter.
    **Storage**: Store at 4°C for up to 1 month.
2. Preparation of washing buffer (complete HBSS, cHBSS):
  a. Prepare the washing buffer containing 0.5% BSA and 2mM EDTA according to the materials table.
    **Storage**: Store at 4°C for up to 1 month.
3. Preparation of 10x Erythrocyte Lysis Buffer (ELB):
  a. Prepare a 10x ELB stock solution according to the materials table.
  b. Filter through a 0.2 μm filter.
    **Storage**: Store at 4°C for up to 1 year.
  c. Dilute to a 1x working solution using distilled water.
    ***Optional:*** Preparation of transcription and translation inhibitors.
    To mitigate *ex vivo* transcriptional changes, prepare stocks in dimethyl sulfoxide (DMSO):
  d. Actinomycin D: 5 mg/mL stock.
  e. Triptolide: 10 mM stock.
  f. Anisomycin: 10 mg/mL stock.
    **Storage:** Use 50 μL aliquots to avoid deleterious freeze-thaw cycles. Store inhibitors at −20°C for a maximum of one month.
    **Warning:** These inhibitors are acutely toxic. Handle them with extreme care by following PPE guidelines and by consulting the Material Safety Data Sheets (MSDS) before use.

### Collection of *post-mortem* human brain tissue

**Timing: 24 h**

This section describes the collection and storage of CNS tissues, CSF, and peripheral blood.

4. Prepare transport tubes:
  a. Fill 50 mL tubes with approximately 25 mL of plain Hibernate A medium.
  b. Pre-weigh the tubes and label them with their weights to facilitate accurate tissue mass calculation during the dissociation steps.
  c. Store prepared tubes at 4°C in the fridge until the autopsy is performed.
5. Tissue sampling and storage:
  a. Place dissected samples of interest directly in plain Hibernate A medium.
  b. Store at 4°C and ensure they are processed within a maximum of 24 h post-autopsy.

***Note:*** Standard yields are typically obtained from the following sample sizes: 2–6 g of parenchyma (subcortical white matter or cortical gray matter), 5–10 cm^2^ of leptomeninges or dura mater, and 0.5–1 g of choroid plexus**.**

6. Cerebrospinal fluid (CSF) collection:
  a. Aspirate CSF from the lateral ventricles using sterile 50 mL syringe.
  b. Transfer the fluid to a 15 mL tube and store at 4°C.

***Note:*** A typical volume is 10 mL of CSF.

7. Peripheral blood collection:
  a. Collect blood via a cardiac puncture into a 6 mL BD Vacutainer® K2E (EDTA) tube.
  b. Store at 20°C.

***Note:*** A typical volume is 5 mL of blood.

**CRITICAL:** It is essential to begin processing all samples as soon as possible following the autopsy. We have established a strict 24-h cutoff for tissue storage in Hibernate A at 4°C. Rapid *post-mortem* autopsies frequently allow for the collection of non-clotted blood containing a high ratio of viable T cells. In our experience, when cell isolation from brain samples is performed more than 36 h (i.e., by the second day) post-autopsy, the cell yield is significantly reduced, although viable cells may still be recovered. However, exceeding these intervals severely impacts the recovery of viable cells and increases the risk of RNA degradation and technical artifacts in downstream application.

### Laboratory preparation

**Timing: 10 min**

8. Pre-warm a water bath at 37°C.
9. Prepare sterilized 100 μm stainless steel mesh cell dissociation sieves, glass Pasteur pipettes, scissors, scalpels, and tweezers.

***Note:*** Sterilize equipment using dry-heat sterilization at 160°C for 2 h. Use extreme caution to avoid cuts or punctures when handling sharp instruments.

10. Thoroughly clean the laminar flow hood with 70% ethanol to ensure a sterile working environment.
11. Prepare a container of ice to ensure all tissue samples and cell suspensions remain at 4°C on ice throughout the processing steps.
  ***Optional:*** If the isolated cells are intended for cryopreservation, place a freezing container (e.g., Mr. Frosty) filled with isopropanol in the refrigerator to pre-chill.

## Key resources table

**Table:** 

REAGENT or RESOURCE	SOURCE	IDENTIFIER
**Antibodies**		

Anti-human CD3–PE-CF594 (1:500 dilution)	BD Biosciences	Cat# 562280 RRID: AB_11153674
Anti-human CD4–R718 (1:100 dilution)	BD Biosciences	Cat# 566931 RRID: AB_2869953
Anti-human CD8–BV510 (1:50 dilution)	BioLegend	Cat# 344732 RRID: AB_2564624
Anti-human CD11b–PE-Cy7 (1:200 dilution)	BioLegend	Cat# 301321 RRID: AB_830643
Anti-human CD45–BB515 (1:100 dilution)	BD Biosciences	Cat # 564585 RRID: AB_2869588
Anti-human CD45RA–BV650 (1:500 dilution)	BioLegend	Cat# 304136 RRID: AB_2563653
Anti-human CD69–BV650 (1:100 dilution)	BioLegend	Cat# 310934 RRID: AB_2563158
Anti-human CD103–PE (1:50 dilution)	BioLegend	Cat# 12-1038-42 RRID: AB_11150242
Anti-human CD197/CCR7–PE-Cy7 (1:10 dilution)	BD Biosciences	Cat# 557648 RRID: AB_396765
LIVE/DEAD Fixable Viability Dye eFluor 780 (1:1,000 dilution)	Thermo Fisher Scientific	Cat# 65-0865
FcR Blocking Reagent	Miltenyi Biotec	Cat# 130-059-901 RRID: AB_2892112

**Biological samples**		

Human brain tissue and blood – 2 Male/2 Female Average Age ± S.D. (years): 74.5 ± 13.2	Netherlands Brain Bank	https://www.brainbank.nl/

**Chemicals, peptides, and recombinant proteins**		

Accutase	Sigma-Aldrich	Cat# SCR005
Actinomycin D	Sigma-Aldrich	Cat# A1410
Actisan (bleach tablets)	VEIP desinfection	N/A
Anisomycin	Sigma-Aldrich	Cat# A9789
Bovine serum albumin (BSA)	Sigma-Aldrich	Cat# 05470-25G
Collagenase IV	Worthington	Cat# LS004189
Distilled water	Thermo Fisher Scientific	Cat# 15230
Dimethylsulfoxide (DMSO)	Sigma-Aldrich	Cat# D8418
DNAse I	Roche	Cat# 11284932001
Dulbecco’s Phosphate-Buffered Saline (DPBS)	Thermo Fisher Scientific	Cat# 14190
Ethanol	Sigma-Aldrich	Cat# 24103-5L-R
Ethylenediaminetetraacetic acid (EDTA)	Sigma-Aldrich	Cat# E5134
Fetal calf serum (FCS)	Thermo Fisher Scientific	Cat# A5256701
Hanks’ Balanced Salt Solution (HBSS), no calcium, no magnesium, no phenol red	Thermo Fisher Scientific	Cat# 14175095
Hibernate A	Thermo Fisher Scientific	Cat# A1247501
Isopropanol	VWR	Cat# 20904.293
Lymphoprep™	STEMCELL Technologies	Cat# 1114544
NaHCO_3_	Merck	Cat# 1.06329
NH_4_Cl	Sigma-Aldrich	Cat# 21330
Percoll™	GE Healthcare	Cat# GE17-0891-01
Triptolide	Sigma-Aldrich	Cat# T3652
UltraComp eBeads Plus Compensation Beads	Thermo Fisher Scientific	Cat# 01-3333-42

**Critical commercial assays**		

RNAeasy Micro Kit	Qiagen	Cat# 74004

**Deposited data**		

Bulk RNA sequencing data	This paper	GEO: GSE307577

**Software and algorithms**		

Bioconductor	Huber et al.^10^	https://bioconductor.org/
biomaRt (version 2.58.2, R package)	Durinck et al.^11^	Bioconductor
edgeR (version 3.42.4, R package)	Robinson et al.^12^	Bioconductor
fastp (version 0.23.2)	Chen et al.^13^	https://github.com/OpenGene/fastp
FastQC (version 0.11.9)	Babraham Institute	https://www.bioinformatics.babraham.ac.uk/
ggplot2 (version 3.5.1, R package)	Wickham et al.^14^	https://CRAN.R-project.org/package=ggplot2
STAR2 (version 2.7.10)	Dobin et al.^15^	https://github.com/alexdobin/STAR
Limma (version 3.56.2, R package)	Ritchie et al.^16^	Bioconductor
PCAtools (version 2.14.0, R package)	N/A	Bioconductor
Flowjo (v10.10.0)	BD Biosciences	https://www.flowjo.com/
HTSeq (version 2.0.2)	Anders et al.^17^	https://htseq.readthedocs.io/en/master/
R (version 4.3.1)	The R Project	https://www.r-project.org/

**Other**		

0.2 μm Syringe filters	Sarsted	Cat# 83.1826.001
4°C fridge/−20°C freezer	Liebherr	Cat# ICNb5123
5 mL serological pipette	Greiner Bio-One	Cat# 606180
10 cm^2^ petri dish	Greiner Bio-One	Cat# 633181
10 mL serological pipette	Greiner Bio-One	Cat# 607180
15 mL conical tubes	Greiner Bio-One	Cat# 188271
25 mL serological pipette	Greiner Bio-One	Cat# 760180
50 mL conical tubes	Greiner Bio-One	Cat# 227270
50 mL syringe	BD	Cat# 309653
70 μm EASYstrainer™	Greiner Bio-One	Cat# 542070
−70°C freezer	Snijders Labs	Cat# HF570
100 μm mesh cell dissociation sieve	Sigma-Aldrich	Cat# CD1-1KT
Aluminium foil	VWR	Cat# 291-0044
BD FACS Aria Cell Sorter	BD Biosciences	N/A
BD Vacutainer® K2E 6 mL	BD	Cat# 367864
Centrifuge	Eppendorf	Cat# 5810 R
Dissecting scissors	Sigma-Aldrich	N/A
Dounce homogenizer	Sigma-Aldrich	Cat# D9938-1SET
Dry-heat oven	Ecocell	N/A
End-over-end mixer	Snijders Labs	Cat# 34528
Freezing Container (Mr. Frosty)	Thermo Scientific™	Cat# 5100-0001
Glass Pasteur pipettes	BRAND®	Cat# BR747725
Laminar flow hood	CleanAir by Baker	BioVanguard
Scalpels	Sigma-Aldrich	N/A
Tweezers	Sigma-Aldrich	N/A
V-bottom 96-well plate	Greiner Bio-One	Cat# 651101
Vortex	Fisherbrand	N/A
Water bath	Lauda	Cat# MA6
Weigh scale	A&D Company	Cat# HR-250AZ

## Materials and equipment

**Table:** Washing buffer (cHBSS)

Reagent	Final concentration	Amount
HBSS, no calcium, no magnesium, no phenol red	N/A	498 mL
BSA	0.5% w/v	2.5 g
0.5 M EDTA	2 μM	2 mL
**Total**	**N/A**	**500 mL**

Store at 4°C for up to 1 month.

**Table:** 10X Erythrocyte Lysis Buffer

Reagent	Final concentration	Amount
NH_4_Cl	1.5 M	8.02 g
NAHCO_3_	100 mM	0.84 g
EDTA	10 mM	0.37 g
Distilled water	N/A	100 mL
**Total**	**N/A**	**100 mL**

Store at 4°C for up to 1 year.

**Table:** Digestion buffer (for white matter)

Reagent	Final concentration	Amount
DNAse I	33 μg/mL	variable
Collagenase IV	300 U/mL	variable
Hibernate A	N/A	20 mL
**Total**	**N/A**	**20 mL**

Use immediately.

**Table:** Digestion buffer (for meninges/choroid plexus)

Reagent	Final concentration	Amount
DNAse I	100 μg/mL	variable
Collagenase IV	300 U/mL	variable
Hibernate A	N/A	20 mL
**Total**	**N/A**	**20 mL**

Use immediately.

## Step-by-step method details

### Tissue dissociation

**Timing: 2 h**

This section describes the dissociation of CNS tissues. The choice between mechanical and enzymatic dissociation depends on the requirement for preserving surface epitopes versus maximizing yield.

**Figure 1:**
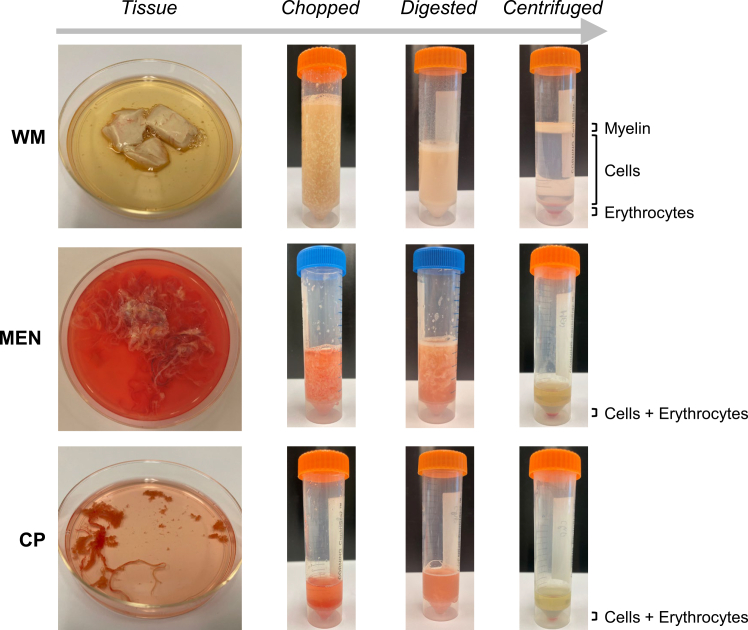
Isolation procedure of central nervous system tissue visualized Illustrated are white matter (WM), leptomeningeal (MEN), and choroid plexus (CP) samples during processing.

1. Calculate tissue mass:
  a. Weigh the pre-labeled tubes containing the brain material and calculate weight of brain tissues.
    ***Note:*** Accurate weighing is critical for calculating the appropriate volume of digestion buffer and inhibitor cocktail.

2. Mince tissues (See Figure 1 for visual guidance):
  a. Choroid plexus, leptomeninges, or dura matter:
    i. Use sterile scissors to cut the tissue as finely as possible.
    ii. Use a scalpel to further mince these tissues smaller.
    iii. Wash these tissues 3 times with 1x DPBS to remove excess blood cells and debris.
  b. White matter:
    i. Remove blood vessels with tweezers.
    ii. Use a scalpel to mince the tissue until it can be pipetted with a 10 mL pipette.
  c. Gray matter:
    i. Use a scalpel to mince the tissue.
    ii. Grinding chopped tissue over a 100 μm mesh cell dissociation sieve using the plunger from a 50 mL disposable syringe.
      ***Note:*** Gray matter is structurally denser than white matter due to a higher concentration of cell bodies and extracellular matrix. This additional mechanical shearing ensures the digestion enzymes can penetrate the tougher cortical tissue efficiently.

3. Transfer the minced tissue into a 50 mL tube and suspend in 20 mL hibernate A medium per 4 g of tissue.
  ***Note:*** For optimal digestion, keep a maximum of 30 mL medium per tube. If the tissue volume is high, divide the suspension across multiple tubes.
  ***Optional:*** Pre-incubation with transcription and translation inhibitors.
  If the isolated cells are intended for next-generation sequencing, it is essential to mitigate heat-induced artifacts caused by the subsequent 37°C enzymatic digestion.
  a. Add inhibitor cocktail: Add the following inhibitors to the tissue suspension:
    i. Actinomycin D: 5 μg/mL.
    ii. Triptolide: 10 μM.
    iii. Anisomycin: 27.1 μg/mL.
  b. Cover the tubes in aluminum foil to protect them from light.
  c. Incubate for 20 min at 4°C using an end-over-end mixer to ensure continuous penetration of the inhibitors into the tissue fragments.
    ***Note:*** This pre-incubation step allows the inhibitor cocktail to penetrate the tissue and halt transcriptional activity before the temperature is raised.
4. Add the enzymes directly to the suspended tissue according to the tissue type:
  a. Choroid plexus, leptomeninges, or dura mater:
    i. Add 300 U/mL collagenase IV and 100 μg/mL DNase I.
  b. White or gray matter:
    i. Add 300 U/mL collagenase IV and 33 μg/mL DNase I.

***Note:*** The higher concentration of DNase I for border tissues helps manage the increased stickiness often encountered with these samples.

5. Vortex each tube for 10 s.
6. Incubate tubes in a 37°C water bath for 60 min and vortex each tube for 10 s every 15 min.

***Optional:*** Alternative Dissociation methods.

**Cold enzymatic digestion (Accutase):** For sensitive transcriptomic studies where heat-induced artifacts must be avoided entirely, tissue can be digested with Accutase at 4°C.

*Trade-off:* This method preserves the transcriptional state without inhibitors but results in significantly lower cell yields.

**Mechanical dissociation (glass dounce homogenizer):** Tissue can be dissociated mechanically to keep the expression of surface proteins entirely intact.

*Trade-off:* This yields the lowest cell recovery but is ideal for markers sensitive to enzymatic cleavage.

***Note:*** Be aware that Collagenase IV can cleave certain surface proteins, rendering them undetectable by flow cytometry. A notable example is CD27. If such markers are critical to your analysis, mechanical dissociation or Accutase should be prioritized.

7. Filter cell suspension through a 100 μm mesh cell dissociation sieve in a 10 cm^2^ petri dish (see Figure 1). This step removes undigested connective tissue and large aggregates.
8. Collect into a new 50 mL tube.
9. Top up the tube to 50 mL with cHBSS.
10. Centrifuge at 600 *g* for 10 min at 4°C.

***Note:*** Aim to process approximately 4 g of tissue per tube. If the sample mass is significantly higher (e.g., 7 g), divide the cell suspension into two separate tubes to prevent clogging and ensure a clean pellet.

***Note:*** Do not fast cool the centrifuge, instead, allow the centrifuge to reach 4°C gradually during the run.

**Safety:** All supernatants and used materials should be discarded into bleach and handled as biological waste in accordance with biosafety regulations.

### Density gradient centrifugation

**Timing: 2 h**

This section describes the removal of myelin and cell debris to obtain a single-cell suspension.

11. Discard the supernatant and resuspend the pellet according to tissue type:
  a. Choroid plexus, leptomeninges, or dura mater: Resuspend in 2 mL cHBSS.
  b. White or gray matter: Resuspend in 20 mL cHBSS.
    ***Note:*** BSA is utilized for density separation as it eliminates the need for myelin removal and results in a higher cell yield, albeit with more debris. The resuspension volume for BSA centrifugation is lower than that for Percoll because BSA separation does not require the formation of distinct density layers for myelin separation.

12. Perform density gradient centrifugation (see Figure 1):
  a. Choroid plexus, leptomeninges, or dura mater (BSA gradient):
    i. Add 5 mL of cold 25% BSA and mix by gentle inversion.
    ii. Centrifuge at 1,200 *g* for 10 min at 4°C (acceleration at 5, brake at 0).
    iii. Carefully remove the supernatant (containing debris) with a pipette.
    iv. Resuspend the pellet in 2 mL cold cHBSS.
  b. White or gray matter (Percoll™ gradient):
    i. Using a pipette, slowly drip 10 mL of cold Percoll™ onto the 20 mL sample.
      **CRITICAL:** Be conscious of time spent dripping Percoll, as it will sediment to the bottom of the tube. Do not mix the Percoll before centrifugation. These issues can result in suboptimal density separation.
      ***Note:*** Maintain a 2:1 ratio of tissue suspension to Percoll (e.g., 20 mL tissue suspension to 10 mL Percoll).
    ii. Centrifuge at 3,100 *g* for 30 min at 4°C (acceleration at 5, brake at 0).
      **CRITICAL:** Do not stop the centrifuge too abruptly, since this will mix the separated layers.
    iii. There are three layers:
      a white myelin layer: on top.
      a transparent layer of mononuclear cells: in the middle.
      an erythrocyte band: on top of the last Percoll™ layer.
    iv. Using a sterilized glass Pasteur pipette, create an opening in the myelin layer and transfer the transparent mononuclear cell layer into a new 50 mL tube.
      ***Note:*** Be careful to not incorporate any myelin or erythrocytes in the mononuclear cell suspension.
      ***Optional:*** Here you can collect the myelin and store it at −70°C for later purification and use.
  c. Top up the tubes with mononuclear cells to 50 mL with cold cHBSS.
  d. Centrifuge at 450 *g* for 10 min at 4°C.
13. Lyse erythrocytes:
  a. Aspirate the supernatant and resuspend cells in 10 mL cold 1X ELB.
  b. Incubate for 5 min on ice.
  c. Top up the tubes to 50 mL with cold cHBSS.
  d. Centrifuge at 450 *g* for 10 min at 4°C.
  e. Aspirate supernatant and resuspend cells in desired volume of cHBSS for further processing.

### Isolation of cerebrospinal fluid mononuclear cells

**Timing: 30 min**

Here we describe the isolation of cells from the CSF.

14. Remove tissue debris from CSF by pouring it over a 70 μm strainer.
15. Spin down CSF at 450 *g* for 10 min at 20°C.
16. Lyse erythrocytes as described before.
  ***Optional:*** The CSF supernatant can be stored at −70°C for later analysis of protein contents.

### Isolation of peripheral blood mononuclear cells

**Timing: 1 h**

This section describes the isolation of peripheral blood mononuclear cells from *post-mortem* blood collected from the heart pouch. This describes the use of Lymphoprep™ according to the manufacturer’s instructions.

17. Mix Lymphoprep™ thoroughly before use by inverting the bottle several times.
18. Add Lymphoprep™ to a tube, according to manufacturer’s instructions.
19. Dilute blood with an equal amount of DPBS.
20. Layer blood on top of Lymphoprep™, being careful to minimize mixing of blood with Lymphoprep™.
21. Centrifuge at 800 *g* for 30 min at 20°C (acceleration at 5, brake at 0).
22. Remove and discard the upper plasma layer without disturbing the plasma:Lymphoprep™ interface.
23. Remove and retain the mononuclear cell layer at the plasma:Lymphoprep™ interface without disturbing the erythrocyte/granulocyte pellet.
24. Wash mononuclear cells once with cHBSS.
25. Lyse erythrocytes as described before.

### Further processing

**Timing: 2 h**

This section describes the preparation of isolated cells for downstream flow cytometry analysis or cell sorting, as performed in Hsiao *et al.*^1^

***Optional:*** If freezing cells, resuspend cells in 1 mL cHBSS, drip 1 mL of freezing medium (80% FCS, 20% DMSO) and freeze in a freezing container at −70°C. Transfer to liquid nitrogen after 24 h.

26. Transfer cells into a V-bottom 96-well plate.
  ***Note:*** Due to the limited yield of T cells from human brain tissue, transfer the entire cell fraction to a 96-well plate in order to ensure optimal yield of viable cells.

27. Block cells with 100-200 μL of 1x FcR-blocking reagent in FACS Buffer (1% BSA and 2mM EDTA in DPBS) for 30 min.
28. Centrifuge at 450 *g* for 10 min at 4°C.
29. Incubate with antibody master mix of choice for 30 min at 4°C. Please refer to Table 1 for possible antibody combinations.

**Table 1:** Specification of antibodies used for cell sorting

Antigen	Company	Clone	Dilution	Fluorochrome
CD3	eBioscience	UCHT1	1:500	PE-CF594
CD4	BD Biosciences	RPA-T4	1:100	R718
CD8	BioLegend	SK1	1:50	BV510
CD11b	eBioscience	B-Ly7	1:200	PE-Cy7
CD45	BD Biosciences	HI-30	1:100	BB515
CD45RA	BioLegend	HI100	1:500	BV650
CD69	BioLegend	FN50	1:100	BV650
CD103	eBioscience	B-Ly7	1:50	PE
CD197/CCR7	BD Biosciences	3D12	1:10	PE-Cy7
LIVE/DEAD Fixable Viability Dye	eBioscience	–	1:1,000	eFluor 780
30. Wash 3 times with FACS buffer by centrifugation at 450 *g* for 3 min and 4°C.
31. Perform flow cytometry analysis or cell sorting.

## Expected outcomes

To optimize cell yields for transcriptomic analysis, we applied enzymatic dissociation within our *post-mortem* human isolation protocols. Previous isolations with mechanical dissociation show a much lower cell yield than with enzymatic digestion (Table 2). Moreover, enzymatic digestion at 37°C with collagenase IV yields superior amounts of viable cells compared to Accutase at 4°C (Table 2).

**Table 2:** White matter cell yield from different enzymatic and mechanical dissociation procedures

Donor	Cells	Col IV 37°C	Col IV + inh. 37°C	Accutase 4°C	Dounce 4°C
1	CD11b^+^ microglia	400,635	348,247	71,168	9,844
	CD69^+^ CD8^+^ T cells	7,271	7,277	989	504
2	CD11b^+^ microglia	124,810	57,162	37,749	7,584
	CD69^+^ CD8^+^ T cells	8,506	3,456	1,476	385
3	CD11b^+^ microglia	32,599	38,027	13,624	3,815
	CD69^+^ CD8^+^ T cells	246	533	140	80
4	CD11b^+^ microglia	464,454	253,160	81,588	68,051
	CD69^+^ CD8^+^ T cells	16,259	9,549	3,190	1,432

3 g per condition, Col IV, collagenase IV; Inh, transcription/translation inhibitors

**Figure 2:**
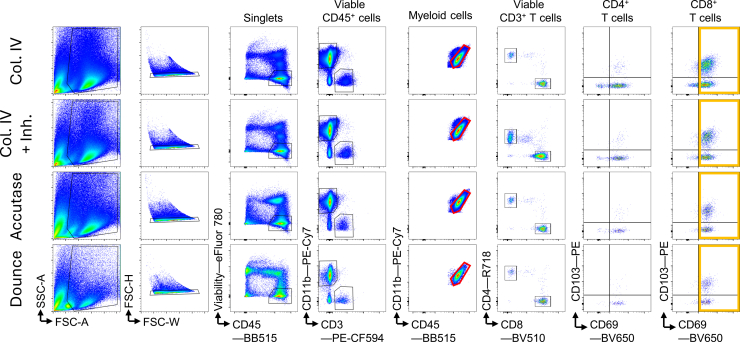
Expected flow cytometry dot plots and gating strategy for sorting From left to right, events are gated on forward and side scatter (FSC/SSC) in order to only include singlets, viable CD45^+^ cells, and subsequently on CD11b^+^ myeloid cells and CD3^+^ T cells. T cells are gated on CD4^+^ and CD8^+^ T cells, and subsequently sorted on the CD69^+^ cell fraction.

White matter T cells are mostly CD69^+^, as can be seen by the dot plots of sorted cells (Figure 2). These dot plots represent the usual phenotype of isolated T cells from different CNS compartments. For researchers interested in other lymphoid populations, it should be noted that we use the same protocol and dissociation parameters for both T- and B-cell isolations.

**Figure 3:**
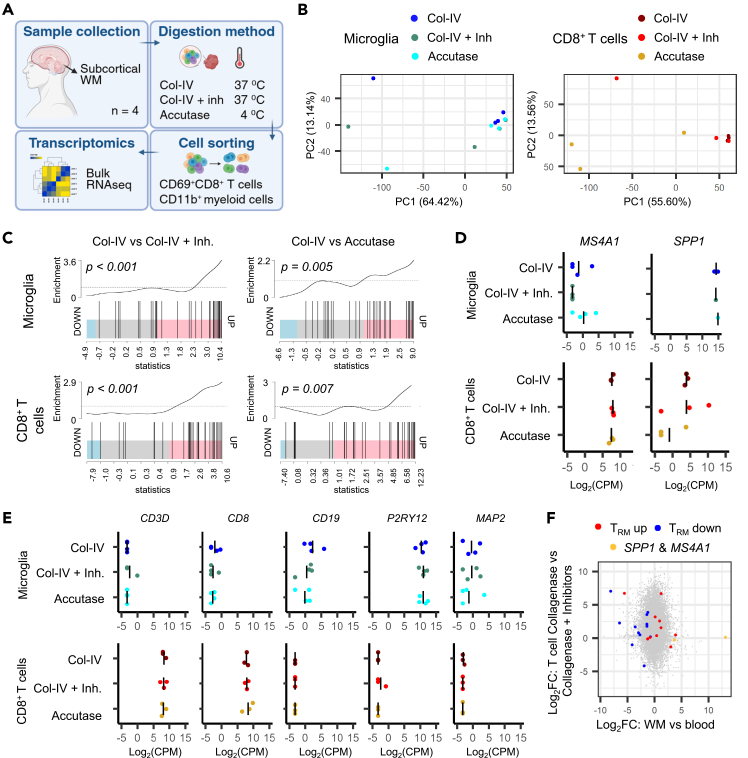
Temperature-induced gene artefacts Bulk RNA sequencing was performed on matched subcortical white matter (WM) CD11b^+^ microglia and CD69^+^ CD8^+^ T cells from n = 4 brain donors. (A) Experimental overview. (B) Principal component analysis (PCA) of microglia and T cells using different dissociation methods. (C) Barcode plot showing enrichment of temperature induced signatures in microglia and T cells following collagenase-IV treatment at 37^0^C without transcription and translation inhibitors. (D) Gene expression of major brain T_RM_-cell markers after different dissociation procedures. (E) Expression of genes associated with T cells (*CD3D*, *CD8A*), B cells (*CD19*), microglia (*P2RY12*), and neurons (*MAP2*) in the sequenced samples, obtained by RNA sequencing. Genes associated with astrocytes (*AQP4*) and oligodendrocytes (*MAG*) were not detected. (F) Log-fold change of WM compared to circulating effector memory T_(EM)_ cells (from Hsiao *et al.*^3^) to the heat-induced signature of T cells. Col-IV, collagenase IV; CPM, counts per million; FC, fold change; Inh., transcription and translation inhibitors; MG, microglia; T, T cell; T_RM_, tissue-resident memory T cell; WM, white matter.

Previous work in animal models by Marsh *et al.* demonstrated that *ex vivo* exposure to elevated temperatures can induce artificial activation signatures in freshly isolated microglia.^9^ To assess potential confounding effects in our workflow, we compared different dissociation conditions by isolating CD11b^+^ myeloid cells and CD8^+^ CD69^+^ T cells from n= 4 subcortical WM samples and performing bulk RNA sequencing (Figure 3A). Across dissociation methods, human microglia and T cells exhibited highly similar enrichment of transcriptomic signatures as found by Marsh *et al.* (Figures 3B and 3C). Key brain T cell characteristics, including *SPP1* and *MS4A1*, as well as purity of samples based on cell type marker genes did not differ between isolation procedures (Figures 3D and 3E). Lastly, the T_RM_ gene signature, remained largely unaffected (Figure 3F).^3^

Based on these findings, we incorporated transcription and translation inhibitors into our isolation protocol for sequencing analysis to further minimize artifactual activation and preserve transcriptional states for downstream analyses. Lastly, the use of this protocol is not limited to T cells and microglia, but can be employed to isolate other mononuclear cells from *post-mortem* CNS tissues. For instance, B cells have been isolated from brain donors with multiple sclerosis using these steps previously.^8^

## Quantification and statistical analysis

Total RNA was isolated with the RNeasy MicroKit according to the manufacturer’s instructions, and RNA sequencing was performed (GenomeScan). All RNA samples were polyA-enriched and then sequenced with a 150-bp paired-end read length on a NovaSeq6000 platform. Quality control was performed using FastQC (version 0.11.9). Adapter sequences were trimmed using fastp (version 0.23.2). Reads were mapped to the human GRCh38.p13 genome using STAR2 (version 2.7.10) on default settings. Feature counts of reads mapped to genes were determined using HTSeq (version 2.0.2). Resulting counts were analyzed separately using R (version 4.3.1) and Bioconductor. Genes with more than 2 counts in a minimum of 2 samples were retained. Count data were transformed to log2-counts per million (logCPM), normalized using the trimmed mean of M-values (TMM) method (edgeR package, version 3.42.4), and precision weighted using voom (limma package, version 3.56.2). The remaining genes were reannotated with the biomaRt package (version 2.58.2), using Ensembl (version 111). PCA was performed on the logCPM values of the 500 most variable genes to distinguish sources of variation using the PCAtools package (version 2.14.0). Differential expression was assessed using an empirical Bayes moderated *t* test within limma’s linear model framework including donor and log10-transformed cell number counts as covariates [Y = ∼0 + experimental condition + donor + log10 (cell number)]. Resulting *p*-values were corrected for multiple testing using the Benjamini-Hochberg false discovery rate (FDR). Genes with FDR <0.05 were considered significantly differentially expressed. Results were visualized with the ggplot2 package (version 3.5.1). Competitive gene set enrichment analysis was performed with CAMERA with preset value of 0.01 for the inter-gene correlation using the Hallmark, C1, C2, C3, C5, C6, and C7 gene set collections retrieved from the Molecular Signatures Database (MSigDB, version 2023.2; https://www.gsea-msigdb.org/gsea/msigdb/index.jsp), heat-induced gene sets were added manually.^9^

## Limitations

There are several limitations to consider for the downstream analysis of primary human brain-resident T cells and microglia. For instance, while the use of enzymatic digestion leads to high yields, it can cleave sensitive surface proteins. This was evidenced in the article associated with this protocol, where CD27 could not be analyzed accurately because it was cleaved during tissue dissociation.^1^ An alternative approach to address this issue is the use of mechanical dissociation using a glass dounce homogenizer, which leads to a lower yield of viable cells and may portray only a robust subset of the total population.

Another critical factor in the recovery of viable cells is the CSF pH upon autopsy.^6^ Therefore, we set a boundary of CSF pH > 6.0 for the use of tissue, which is assessed during the “before you begin” stage. This threshold is based on previous research indicating that lower pH values correlate with poor tissue quality and reduced cell viability.

Furthermore, if freezing cells for later analysis, the use of transcription and translation inhibitors harshly affects the viability of frozen cells. Therefore, it is important to process cells for sequencing approaches directly after isolation. Lastly, if the aim of the isolation is to culture primary cells, a limitation can be potential contamination, since *post-mortem* brain tissues are not sterile at autopsy. While we do not perform routine contamination testing due to the short-term nature of our cultures and the high value of the tissue, users should monitor for signs of microbial growth if long-term applications are intended.

## Troubleshooting

### Problem 1

Low cell yields from tissue samples (related to step 31).

### Potential solution

Screen donors based on CSF pH, as viable cell recovery is highly correlated with pH levels.^6^ We recommend an inclusion threshold of pH > 6.0 to ensure sufficient cell viability and transcriptional integrity.

### Problem 2

Excessive debris in white matter samples (related to step 12b).

### Potential solution

Always pass the cell suspension through a filter before using a flow cytometer. Another option is that too much myelin was taken up after density centrifugation. In that case, a Percoll™ density gradient can be performed again after visual inspection of the pellet.

### Problem 3

Poorly defined or disrupted Percoll™ layers (related to step 12b).

### Potential solution

The integrity of the myelin layer can differ vastly between brain donors based on diagnosis or degree of brain atrophy. To ensure better delineation, ensure the Percoll™ is dripped slowly to maintain the 2:1 ratio. If the integrity of the myelin layer is low, use extreme caution with the Pasteur pipette to avoid accidental uptake of myelin into the mononuclear cell fraction.

***Note:*** We observe no issues that are inherently due to the density gradient itself; however, the physical characteristics of the donor tissue are the primary drivers of layer definition. If layers are poorly defined, a second centrifugation step may be required.

## Resource availability

### Lead contact

Further information and requests for resources and reagents should be directed to and will be fulfilled by the lead contact, Cheng-Chih Hsiao (c.hsiao@nin.knaw.nl).

### Technical contact

Technical questions on executing this protocol should be directed to and will be answered by the technical contact, Cheng-Chih Hsiao (c.hsiao@nin.knaw.nl).

### Materials availability

This study did not generate new unique reagents.

### Data and code availability

The dataset generated during this study is available at GEO: GSE307577.

## Acknowledgments

We extend our sincere gratitude to the donors and their families for their invaluable contribution to research through the Netherlands Brain Bank. We thank the Netherlands Brain Bank staff for their expertise in performing the autopsies and providing logistical support. We acknowledge the Flow Cytometry Core Facility at Amsterdam 10.13039/501100003761UMC for technical assistance and access to sorting equipment. We also thank Dr. Michael Mingueneau and Dr. Lasse Dissing-Olesen for their insightful suggestions. This work was supported by the Nationaal MS Fonds (OZ2018-003).

## Author contributions

Conceptualization, J.S., J.H., and C.-C.H.; methodology, H.J.E. and C.-C.H.; investigation, H.J.E. and C.-C.H.; writing – original draft, H.J.E. and C.-C.H.; writing – review and editing, I.H., J.S., J.H., and C.-C.H.; funding acquisition, I.H., J.S., and J.H.

## Declaration of interests

The authors declare no competing interests.
